# Immunotherapy and Allogeneic Bone Marrow Transplantation in B Acute Lymphoblastic Leukemia: How to Sequence?

**DOI:** 10.1007/s44228-022-00006-6

**Published:** 2022-05-11

**Authors:** Anna Komitopoulou, I. Baltadakis, I. Peristeri, E. Goussetis

**Affiliations:** 1 Stem Cell Transplant Unit, “Agia Sofia Children’s Hospital”, Mikras Asias 46 and Levadias 8, 11527 Athens, Greece; 2 Department of Haematology and Bone Marrow Transplantation Unit, Evangelismos Hospital, Athens, Greece

**Keywords:** Immunotherapy, B-ALL, Therapeutic choice

## Abstract

Long-term disease control is achieved in 80–90% of patients with acute lymphoblastic leukemia of B origin (B-ALL). About half of adult and 10% of pediatric patients develop refractory or relapsed disease, whereas survival after relapse accounts about 10% in adults and 30–50% in children. Allogeneic bone marrow transplantation offers remarkable benefit in cases with unfavorable outcome. Nevertheless, novel immunotherapeutic options have been approved for patients with adverse prognosis. Immunotherapeutic agents, nowadays, are preferred over standard chemotherapy for patients with relapsed or refractory B-ALL The mode of action, efficacy and safety data of immunotherapeutic agents released, indications and sequence of those therapies over the course of treatment, are herein reviewed.

## Introduction

Acute lymphoblastic leukemia of B origin (B-ALL) is equally aggressive in adults and children. Long-term disease control with further consolidation and maintenance is achieved in 80–90% of patients; nevertheless, about half of adult and 10% of pediatric patients develop refractory or relapsed (r/r) disease. Survival after relapse (5-year survival) is dismal at about 10% in adults and 30–50% in children. Allogeneic bone marrow transplantation offers remarkable benefit in patients with r/r B-ALL [1–3]. Currently, new immunotherapeutic options have gained approval for patients with adverse prognosis. In view of the availability of innovative therapies, the choice of the most appropriate therapeutic option for a patient with B-ALL constitutes a great challenge [4, 5].

## Minimal/Measurable Residual Disease (MRD)

Relapses mainly occur because leukemic cells still remain in the body, even after complete hematologic response has been achieved. The term MRD is used to describe the low-level disease which is not detectable by conventional cytomorphology [6, 7]. MRD positivity or reappearance after initial response is defined as molecular failure or relapse, and is an indication of impending hematologic relapse. Early MRD negativity defines a group of patients with prompt leukemic blast clearance and low probability of relapse. On the other hand, MRD positivity after the maintenance phase of treatment may point to an upcoming relapse and, thus, salvage treatment has to be initiated the soonest [6–12]. MRD high-risk patients are shown to benefit from allogeneic hematopoietic stem cell transplantation (HSCT), which adds to the improvement of response duration and survival. Unfortunately, allogeneic HSCT is not often an option for specific patients having comorbidities or for those with early disease relapse, not achieving second remission [7–11]. In addition, the prognostic relevance of pre-transplantation MRD positivity is not a marker of favourable outcome after transplant. Achievement of hematologic remission of r/r patients, but also MRD positivity conversion to negativity is the therapeutic goal of novel immunotherapy agents [13, 14].

## New Immunotherapy Agents

Overt hematologic relapse and MRD are the target of novel immunotherapeutic agents and monoclonal antibodies, such as blinatumomab and inotuzumab ozogamicin, but also of cellular therapies like chimeric antigen receptor T cells (CAR T cells) [4, 5]. Moreover, the use of MRD as an index for guiding clinical decisions in current treatment protocols, as a condition for approval of novel therapies, generates the need for redefinition of the role of HSCT in B-ALL [1, 2, 15].

### Blinatumumab

Blinatumomab is a recombinant murine monoclonal bi-specific antibody that is CD19 directed with CD3 T-cell engagement, promoting immune-mediated elimination of B-cell lymphoblasts by cytotoxic T cells. In a phase II clinical trial which was conducted by GMALL group, blinatumomab was administered as a 4-week continuous intravenous infusion at a dose of 15 μg/m^2^/24 h in 21 patients with MRD persistence (≥ 10^–4^ threshold) after consolidation. In patients with an allogeneic donor, an HSCT was offered at any time after the first cycle of treatment with blinatumomab. Responders were permitted to receive three additional consolidation cycles of treatment with blinatumomab. Molecular remission was achieved in 80% of patients who were not MRD negative at any time before blinatumomab initiation. According to a more recent analysis of the trial with a median follow up of 51 months after blinatumumab, half of the patients remained in remission (10/20), whereas almost 50% of them, had not been transplanted. So, it seems that there may be a possibility of long-term complete remission (CR) in patients with chemo-refractory disease after therapy with blinatumomab, even without HSCT [16, 17].

In the BLAST clinical trial (NCT01207388), blinatumomab was investigated in patients with B-ALL in first or second CR having MRD ≥ 0.1% Among the 113 patients included, 78% (*n* = 88) achieved MRD negativity after one cycle of therapy, and 67% of them proceeded to HCT. The relapse-free survival (RFS) and overall survival (OS) were significantly prolonged in MRD responders (23.6 versus 5.7 months, *P* = 0.002 and 38.9 versus 12.5 months, *P* = 0.002, respectively). Complete MRD response rates were similar between patients with MRD ≥ 10^–2^ and those with MRD < 10^–2^ at baseline, and also between patients with first and those with later remission at baseline. In a median follow-up period of 30 months, median RFS and OS were found to be 18.9 months (95% CI range 12.3–35.2) and 36.5 months (95% CI range 19.8–not estimable), respectively. MRD response after the first cycle resulted in better OS and RFS. Seventy-four (67%) of 110 patients underwent HSCT while being in continuous remission after blinatumomab. Nine (25%) of 36 patients without HSCT or chemotherapy after blinatumomab remained in continuous CR, in a median follow-up of 24.0 (range 2.8–41.6) months, whereas 36 (49%) of 74 with HSCT remained in remission. So, patients with a complete MRD response who remained in long-term remission without subsequent HSCT were identified, confirming the results of the pilot study indicating long-term survivors without subsequent HSCT. Transplantation offers an advantage in patients transplanted beyond a second remission [18, 19].

In summary, according to the BLAST trial results, among patients with chemotherapy-resistant MRD, targeted immunotherapy with blinatumomab resulted in a substantial molecular response rate and improved long-term outcomes among responders compared with non-responders. Targeted treatment in early stages of MRD is considered a viable therapeutic strategy for patients with B-cell precursor ALL. The role of HSCT after blinatumomab therapy has not yet been clearly determined, since patients with MRD response after blinatumomab may achieve long term disease control even without allogeneic HSCT. Specifically, according to the updated results of the BLAST trial with a median follow up of 5 years, 40% of the patients being transplanted (*N* = 74) remain in CR, whereas 19% of those not transplanted (*N* = 36), also remain in CR without subsequent HSCT. In addition, among patients with complete MRD response, 46% of patients being transplanted and 30% of those not transplanted remain alive in CR. So, 30% of patients without detectable MRD after blinatumomab administration who do not proceed to transplant, can achieve long term remission [17–20].

A multi-institutional phase-3 trial, assigned adults with heavily pre-treated relapsed or refractory B-cell precursor ALL to receive either blinatumomab or standard-of-care chemotherapy. Blinatumomab was associated with significantly higher remission rates (both CR and CR with incomplete hematologic recovery-CRi) (44% versus 25%; *p* < 0.001), longer median duration of remission (7.3 months versus 4.6 months) and OS (7.7 months versus 4 months; *p* = 0.01) compared with the conventional chemotherapy group. Patients with < 50% blasts in the bone marrow compared to those with ≥ 50% had a twofold probability of achieving CR. This trial confirmed the results of the multicenter phase II study MT103-211 in adult patients with r/r B-ALL which resulted in the Food and Drug Administration (FDA) granting accelerated approval for blinatumomab in December 2014 [4, 5, 19, 21]. The blinatumomab FDA approval was expanded in 2017 to include adult patients with Philadelphia chromosome positive (Ph+) B-ALL based on the results of the ALCANTARA (NCT02000427) clinical trial. This latter trial enrolled 45 patients with R-R or intolerance to tyrosine kinase inhibitors. MRD negativity was noted in 88% of responders; and 44% of them proceeded to HSCT [22].

Additionally, activity of a frontline approach based on dasatinib plus steroid administration as induction treatment followed by the infusion of blinatumomab in adult Ph + ALL has been explored in the D-ALBA trial [23].

Blinatumumab administration is equally effective in pediatric patients with chemo-resistant or r/r B-ALL [24–26].

The toxicity of blinatumomab administration is mainly cytokine release syndrome (CRS) and neurotoxicity. Severe CRS is quoted at about 2–5% and ≥ grade III neurotoxicity about 7–13%. Treatment strategies include corticosteroids or temporary discontinuation of infusions. Permanent discontinuation is considered in higher grade CRS or neurotoxicity with life-threatening complications [17–19, 21, 27].

### Inotuzumab Ozogamicin

Inotuzumab ozogamicin is a CD22 directed antibody bound to the cytotoxic antitumor antibiotic calicheamicin. In August 2017, the FDA approved inotuzumab ozogamicin for the treatment of adult patients with r/r B-ALL. The approval was based on data from the INO-VATE trial [28–30]. In this randomised trial, 326 patients participated. Rates of CR were significantly higher with inotuzumab compared with standard therapy (80.7% versus 29.4%, *p* ≤ 0.001). Among the patients who achieved CR, a higher percentage in the inotuzumab ozogamicin group compared to the standard therapy had results below the threshold for MRD (0.01% marrow blasts) (78.4% versus 28.1%, *p* ≤ 0.001), respectively. In addition, the rates of CR or CRi associated with inotuzumab ozogamicin were similar among patients with high (> 50% blasts) and those with low disease burden, as assessed by the percentage of bone marrow blasts at baseline [29–32].

Inotuzumab ozogamicin in combination with low intensity chemotherapy (mini-HCVD) with or without blinatumomab has been used as frontline therapy for older patients with Philadelphia chromosome-negative ALL. The 3-year event-free survival rate for patients who received HCVAD and those who received the combination of inotuzumab ozogamicin plus mini-HCVD with or without blinatumomab was 34% and 64%, respectively (*p* = 0.003), and the 3-year OS rates was 34% and 63%, respectively (*p* = 0.004) [33].

The above combination has been also used as salvage therapy in r/r Philadelphia chromosome-negative ALL. Overall, 80% of patients responded, whereas 57% achieved CR. The overall MRD negativity rate among responders was 83% [33].

Subsequent remission consolidation with allogeneic HSCT after achieving CR in the inotuzumab group was one of the predictors of better OS. Factors associated with improved OS in the inotuzumab arm were: best MRD status, baseline platelet count and hemoglobin level, duration of first remission, achievement of CR/CRi, as well and whether a patient underwent HSCT during follow‐up. Inotuzumab ozogamicin is effective in extramedullary disease and is related to veno-occlusive (VOD) disease in patients receiving HSCT, especially in those receiving multiple alkylating agents [1, 2, 28–33].

Currently, combination of inotuzumab with BCL-2 inhibitors is under investigation in r/r B-ALL [34].

Hepatotoxicity in the form of hyperbilirubinemia, transaminitis, and sinusoidal obstruction syndrome (SOS, also called VOD) has been seen consistently with inotuzumab. In the INO-VATE trial, 22% of patients undergoing HSCT and 8% not undergoing HSCT presented with SOS. The mechanism of hepatotoxicity with inotuzumab is likely a result of the calicheamicin component of the drug. Preventive strategies should be considered, such as avoiding double alkylators with HSCT following inotuzumab or avoiding more than two cycles of inotuzumab if HSCT is planned [27, 29–32].

### Chimeric Antigen Receptor T Cells (CAR T cells)

Tisagenlecleucel (tisa-cel) was approved by the FDA in August 2017 as the first CAR T cell agent for pediatric and young adult patients (up to 25 years of age) with r/r B-ALL, based on the results of the ELIANA study (NCT 02435849). Patients (*n* = 75) included in the study represented a very high-risk population. They all had chemorefractory disease with extensive bone marrow infiltration and 61% of them had undergone allo-HSCT. The CR rate at 3 months was 82% among infused patients, with negative MRD in 98%. The OS at 24 months reached 66%. Durable remission was observed in 62% of responders at 24 months from infusion [35–37]. Real-world experience with commercially available tisagenlecleucel was reported by the Centre for International Blood and Marrow Transplant Research (CIBMTR). Results from 255 patients included in the study were similar those in the ELIANA trial, with a response rate of 85.5%, 12-month duration of response (DOR) 61%, and 12-month OS of 77.2%. Efficacy was not influenced by previous administration of blinatumomab or central nervous system (CNS) involvement. Patients who were MRD negative before tisa-cel infusion, had OS of 96% at 6 months and low rate of toxicity [38]. In a recent trial, the determinants of CD19 (−) relapse after tisa-cel infusion in pediatric patients and young adults were high tumor burden (occurrence of CRS), prior blinatumomab, detectable MRD at Day 28 (Sub-distribution hazard ratio (SHR) 7.2, *p* = 0.006), whereas CD19 + relapse correlated with loss of B-cell aplasia (BCA) (SHR 21.7, *p* = 0.004) [39].

Brexucabtagene autoleucel (KTE-X19) was recently approved by the FDA for adult patients with r/r ALL based on the results of the ZUMA-3 trial. The Phase 1 part of the study examined three dose levels, and the dose of 1 × 10^6^ cells per kg was selected for the subsequent phase 2 study based on efficacy and safety. The Phase 2 ZUMA-3 study enrolled 71 patients, older than 18 years, with morphological disease in the bone marrow (blasts > 5%) at study entry. The ZUMA-3 study represents the largest adult-only population treated for R/R B-ALL to date [40].

At a median follow-up of 16.4 months, 71% of treated patients achieved CR or CRi, with 56% of reaching CR and 97% of responders having MRD negativity. The median DOR was 12.8 months regardless of censoring patients at subsequent allo-HSCT.

At data cutoff, 12 (31%) of the 39 patients with CR or CRi were in ongoing remission without subsequent allo-HSCT. Median RFS was 11.6 months in all treated patients and 14.2 months in responders. Median OS was 18·2 months in all treated patients and was not reached in responders [40].

In another trial in adult patients, clearance of the leukemic clone by High-Throughput Sequence (HTS) after CD19 CAR T cells was associated with better DFS and OS. Also, in univariable analysis, allogeneic HSCT after CART-cell therapy was associated with longer event free survival (EFS) compared with no allogeneic HSCT (HR 0.31; 95% CI 0.13–0.79; *p* = 0.014) [41]. An additional trial showed that the pretreatment disease burden was a useful predictor of remission duration and survival [43]. Ongoing trials with tisagenlecleucel are being investigated for the treatment of adult patients with ALL (OBERON NCT03628053, phase III, open-label, multinational randomized trial using tisagenlecleucel versus blinatumomab or inotuzumab in adults with r/r B-ALL), and upfront post consolidation phase of therapy for very high-risk pediatric patients with ALL (CASSIOPEIA NCT03876769) [1, 2, 44].

While CAR T-cells are rationally designed targeted therapies, nevertheless they frequently induce life-threatening toxicities. The most common are CRS and immune effector cell-associated neurotoxicity syndrome (ICANS), while other adverse events such as cytopenias, hypogammaglobulinemia, tumor lysis syndrome or infusion reactions may occur after CAR T cell infusion and need to be taken into account in clinical practice. Grade III–IV adverse events occur more frequently with CAR T cell therapy than blinatumomab [1, 2, 15, 45–47].

Differences in the study population between the pivotal trials leading to approval of the above-mentioned immunotherapeutic agents are depicted in Table 1. It has to be stated that no randomized head-to head trials comparing the effects of these immunotherapies have yet been conducted. Pivotal trials have substantial differences in design and patient characteristics, which make direct comparisons of treatment efficacy challenging [4, 5, 16–19, 30, 35, 48–50].

**Table 1:** Pivotal studies for inotuzumab ozogamicin, blinatumomab, tisagenleucel (without matching-adjusted indirect comparison analysis) [5, 21, 29, 30, 35, 36]

	Inotuzumab ozogamicin	Blinatumomab	Tisagenleucel
Pivotal trial	INO-VATE ALL (Phase III)	TOWER (phase III)	ELIANA (phase II)
Target	Anti-CD22 antibody conjugated to calicheamicin	Bispecific antibody that is CD 19 directed with CD3 T-cell engagement	Autologous T-cells with transgene encoding chimeric antigen receptor targeting CD19-positive B cells
Number of patients studied	326 (218 used in primary analysis)	405 (376 received treatment)	92 (75 used in primary analysis)
Comparison group	Salvage therapy such as FLAG, cytarabine, mitoxantrone, high-dose cytarabine	Salvage therapy such as FLAG with or without anthracycline, high-dose cytarabine, methotrexate and clofarabine based regimens	None
CR	80% vs. 29%	44% vs. 25%	81%
MRD negative status (among patients who achieved CR)	78% vs. 28%	76% vs. 48%	100%, 95% by day 28
Duration of remission	4.6 vs. 3.1 months	7.3 vs. 4.6 months	Not reached
Median PFS	5.0 vs. 1.8 months	31% vs.12% EFS at 6 months	73% at 6 months; 59% at 12 months
Median overall survival	7.7 vs. 6.7 months	7.7 vs. 4.0 months	19.1 months
Grade 3 adverse events	91% vs. 95%	87% vs. 92%	88%

*CR* complete remission with or without complete hematologic recovery, *MRD* minimal residual disease, *PFS* progression free survival

## CAR T Cell Therapy Limitations: Consolidation with Allogeneic HSCT Transplantation?

Two distinct types of relapse are recognized: antigen positive versus antigen negative (including lineage switch). Lack of persistence of circulating CAR T cells, often coupled with the loss of BCA, is associated with antigen-positive relapses.

Factors influencing the persistence of CAR T cells include the quality of T cells collected during leukapheresis, CAR design (CD28 < 4-1BB), and the tumor or antigen burden. In fact, loss of BCA is a surrogate marker of CAR-T cell persistence. Early loss of BCA (< 6 months from infusion) correlates with relapse. Patients with CAR T cell persistence are in risk of CD19-negative relapses. The mechanism of relapse is considered to be the downregulation or loss of target epitope, commonly referred to as antigen escape. The underlying mechanism is the selection for pre-existing alternatively spliced CD19 isoforms. The ELIANA trial reported that 15 of 22 relapsed patients experienced a CD19-negative relapse [1, 2, 35, 36, 44, 49, 51].

The major risk factor for CD19-relapse is high tumor burden, which can result in larger clonal heterogeneity due to the emergence of antigen-negative clones after therapy.

Lineage switch from a lymphoid to myeloid origin, with loss of CD19 epitope is another form of antigen escape, often seen in patients with MLL gene rearrangement [44, 49, 51–53].

Prior therapy with blinatumomab poses a potential risk for antigen escape after CD19-specific CAR T cell therapy. Strategies to improve outcome and prevent relapses after CD19 CAR T cell therapy include the use of bivalent or bitransduced CD19 + /CD22 + CAR T cells, the use of humanized CAR T cells to prevent immune rejection and the combination with immune check point inhibitors (f.ex tisa-cel with pembrolizumab or nivolumab). Recently, “armored” CAR T cells that express CD40 ligand, secretable cytokine IL-18, or secretable PD-1 blocking single-chain variable fragment (scFvs) can augment the efficacy of the modified cells [1, 2, 15, 44, 46].

Whether allogeneic transplantation should be initiated in patients after CAR T cell therapy is still a matter of debate [1, 2, 15, 20, 44]. It depends on the CAR T cell construct used, on patient performance status or on whether the patient had received lymhodepletion before CAR T cell infusion. An important parameter is the duration of BCA and of MRD response by Next-Generation Sequencing (NGS). Specifically, patients who lose BCA within less than 2 months after infusion are at greater risk of relapse. In addition, according to the ELIANA trial, patients with no detectable MRD on D28 bone marrow by NGS had superior outcomes. Another issue that has to be taken into consideration is whether a patient has already undergone HSCT and, if so, whether the interval from HSCT to relapse is longer or shorter than 12 months. Also, the possibility of CD19-negative relapse (ex MLL-rearranged B-ALL, prior blinatumomab administration) favors consolidation with bone marrow transplantation. Allogeneic HSCT after CAR T-cell therapy in adult patients was found to be associated with longer EFS compared with no allogeneic HSCT (Hazard ratio (HR) 0.31; 95% CI 0.13–0.79; *p* = 0.014) in patients with high-risk features such as those with high pre lymphodepletion lactate dehydrogenase concentration (HR 1.38 per 100 U/L increment increase), higher pre lymphodepletion platelet count (HR 0.74 per 50 000/mL increment increase) and those who did not have fludarabine incorporated into the lymphodepletion regimen [1, 2, 15, 20, 37, 44, 48, 49].

The rate of graft versus host disease (GVHD) does not seem to increase in patients who receive HSCT after CAR T cell infusion [1, 2, 44, 48, 49].

### Choice of Immunotherapy

#### Blinatumumab Versus Inotuzumab

According to the pre-mentioned data regarding efficacy and limitations of each immunotherapy it could be suggested that inotuzumab and blinatumomab may act as a bridge to transplant, although both can be used as single agents in patients unsuitable for transplant. Blinatumumab can be offered as a definite treatment for long term disease control, while inotuzumab may be limited to two cycles in transplant-eligible patients. Blinatumumab can also be used in patients who achieve MRD-positive disease after inotuzumab. Inotuzumab is effective regardless of age, but adverse effects above grade III are less tolerated in patients over 55 years old. Blinatumumab can also induce good responses in older patients or those with multiple comorbidities, but adverse events may be less tolerated in older patients. Patients with high disease burden may get a higher benefit with inotuzumab. On the contrary, blinatumomab is preferred in low tumor burden disease, with excellent responses in MRD-positive cases. Patients with < 50% blasts in the bone marrow have better CR/CRi rates compared to those with > 50% of blasts [4, 5, 15, 16, 18, 30, 45].

### Choice According to Toxicity

CAR T cells are effective regardless of disease burden [41]. However, higher tumor burden may be correlated with more serious CRS. Toxicities related to blinatumomab seem to be higher among patients with higher tumor-burden. Inotuzumab may lead to VOD, especially in patients subsequently undergoing HSCT [4, 5, 27–30]. In addition, patients preferring to avoid HSCT may be better served with CAR T-cell therapy.

The group of transplant ineligible patients may include older adults, those with multiple comorbidities, or decreased performance status. Inotuzumab may be an effective option in these patients. With a lower risk of VOD without HSCT, inotuzumab may be used for a total of six cycles. Because it can be used in an outpatient setting, it may be especially attractive in transplant ineligible patients who desire to avoid hospitalization. Blinatumumab is also effective in this group of patients, but only a small proportion of may achieve long-term disease control without HSCT [27, 45, 47].

### Novel Immunotherapies and HSCT

MRD-negative status, T-cell expansion and B cell depletion are associated with longer survival in patients receiving CAR T cell therapy. This may be considered a definite treatment without subsequent transplant in patients who achieve MRD negative CR and persistent B-cell aplasia. HSCT can be used as a consolidation therapy in some patients, particularly those not achieving MRD negative CR after CAR T cell therapy. The rate of relapse in ALL after HSCT is reported to be 26–64% [4, 5, 14, 20, 40]. The INO-VATE trial included 17 patients with prior use of HSCT. Inotuzumab resulted in CR in 76% of those patients. Blinatumomab in patients who relapsed after HSCT was associated with a CR/CRi rate of 45%. Nevertheless, in patients who have not undergone previous HSCT and have a suitable donor, HSCT may be preferable to CAR T cells, as there is not yet a long follow-up so as to estimate treatment efficacy of CAR T cell therapy without HSCT consolidation, and the cost of the therapy is also a limitation. Although there may be a benefit of allografting in selected refractory patients not in remission, it should be preferably performed after achieving remission first [4, 5, 15, 20, 44, 48, 49].

Blinatumomab is currently being studied as post-transplant maintenance in a phase II trial (NCT02807883) and is the only agent currently approved for MRD + disease. However, the type of patients who may not need HSCT and may achieve long term disease control with blinatumomab alone is unknown. Nonetheless, if there is a high possibility to undergo CAR T-cell therapy in the future, there is a small risk of loss of CD19 expression with blinatumomab [5, 6, 15, 44, 49].

### Immunotherapy in Relapsed/Refractory Ph + B-ALL

Blinatumumab overcomes T315I mutation-induced resistance and, as a single agent, is effective in patients who have failed ponatinib. It is often combined with tyrosine kinase inhibitor (TKI) in r/r Ph + ALL. Also, out of 38 Ph + patients from the INO-VATE trial and a phase I/II trial who failed prior TKI ± HSCT, 66% achieved complete remission, and 63% had MRD negativity. An ongoing phase I/II trial is studying the combination of inotuzumab with bosutinib for patients with R/R Ph + ALL (NCT02311998) [5, 22, 23].

### Trials in Progress

Several other trials regarding novel immunotherapies are in progress, such as a phase I study with blinatumomab in combination with checkpoint blockade with nivolumab and ipilimumab (ClinicalTrials.gov NCT02879695), a phase III study investigating the role of blinatumomab combined with chemotherapy in patients with standard-risk B-ALL, a single-arm phase I/II study (ZUMA-4) which evaluates the safety and efficacy of brexucabtagene autoleucel in pediatric patients with B-ALL that is refractory, relapsed after at least one salvage therapy, or relapsed after HSCT (ClinicalTrials.gov NCT02625480). Additionally, a phase 3 study will be investigating the role of inotuzumab ozogamicin combined with chemotherapy in patients with newly diagnosed high-risk B-ALL, and an ongoing clinical study (NCT03628053) is comparing tisagenlecleucel versus blinatumomab or inotuzumab in r/r ALL [1, 2, 15, 20, 31, 44, 48–51].

### Choice and Limitations Among Available Immunotherapies

Despite the success with improved outcomes in patients with B-ALL over recent decades, risk factors in a subset of cases continue to suggest poor prognosis. Review of risk stratification has led to the practice of augmenting treatment intensity for patients who are at high risk of failure or relapse. The current approach for patients with de novo high-risk ALL is to augment the intensity of chemotherapy. Certain high-risk subgroups, such as those with Ph+ ALL, may benefit from additional drugs (i.e., TKIs). HSCT after induction of remission in certain very high-risk subgroups is an option, especially if a suitable matched related donor is available. Although augmented chemotherapy and HSCT may lead to long-term cure in some of these patients, many relapse or have disease progression. Currently, immunotherapeutic options are better than chemotherapy for achievement of MRD-negative disease in r/r B ALL patients, while there is lack of safety data from head to head comparison between these options [4, 5, 15, 44, 53, 54].

Choice between these therapies depends on several factors, such as donor availability, the presence of CD19 + or CD22 + on blast cells, tumor burden, toxicity, previous therapies. Combination and sequence of these therapies remain a challenge. Specifically, patients who remain MRD positive after inotuzumab, can be rescued by blinatumomab administration and vice versa. CAR T cell therapy can augment duration of remission in patients who have received inotuzumab or blinatumumab or a combination of both. Allogeneic HSCT may serve as consolidation after CAR T infusion. On the other hand, CAR T cell therapy can be used as a bridge to transplant. Second transplantation in patients who reach long term remission after CAR T cell infusion may lead to more toxicity than efficacy. It still remains a topic of discussion whether CAR T cells can be the final answer for r/r B ALL. Furthermore, cellular therapy may be an alternative in patients with Down or Li-Fraumeni syndromes who suffer from notable toxicity from chemotherapy and conditioning regimens [4, 5, 15, 44, 48, 51, 53, 54].

In addition, treatment of r/r ALL is a challenge in patients with central nervous system (CNS) infiltration for which there is no unified treatment regimen. Combination of intrathecal and conventional chemotherapy is mainly used to achieve CR. CD-19 CAR-T cells have a beneficial effect on patients with active CNS disease, as they cross the blood–brain–barrier (BBB). Inotuzumab ozogamicin also has a positive effect on r/r B-ALL with CNS involvement [4, 5, 15, 44, 53–55].

A limitation of CAR T cell therapy is the cost. It is an expensive treatment option and presents a challenge for insurers and health care systems. The price list for tisagenlecleucel is 475,000 USD for a single infusion and that amount does not take into account the costs of supportive care, tocilizumab for patients who develop CRS, or other treatments which may be necessary to maintain a durable remission (e.g., HSCT) [54, 56]. Recent data demonstrate that blinatumomab is cost-effective compared to standard chemotherapy for children with high-risk relapse of B-ALL [57].

Choice of immunotherapy is summarized in Tables 2 and 3. Mechanisms of immunotherapy failure include loss or decreased expression of CD19 or CD22, the (4; 11) chromosomal translocation, acquired mutations, myeloid switch, and lack of expansion or persistence of CAR T cells [4, 5, 15, 20, 44, 48–51].

**Table 2:**
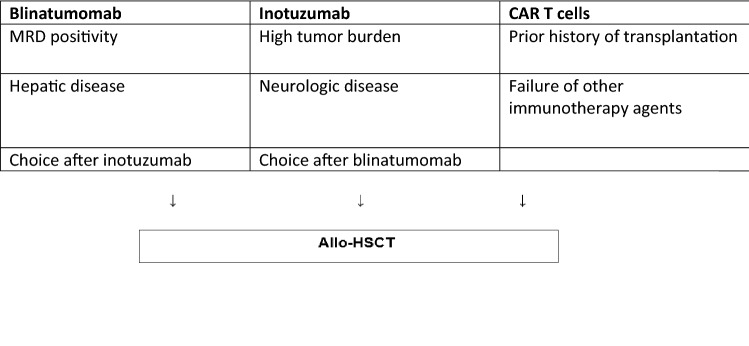
Choice of immunotherapy [4, 5, 15, 44, 49, 53, 54]

**Table 3:** Choice of immunotherapy

Inotuzumab	Indicated in patients with high disease burden (≥ 50%) blast cells and/or extramedullary disease
Blinatumomab	Indicated in patients with MRD( +) disease while in morphologic CR, eligible or not for allogeneic bone marrow transplantation Indicated in patients in relapse with low disease burden (< 50%blast cells)
CARTs	Achievement of deep complete responses in 80% of patients Response in extramedullary disease FDA and EMA approved for pediatric and young adult patients (up to 25 years of age) with r/r B-ALL Limitation: time-consuming process Final answer? No need for transplant?

Therapeutic management of relapsed/refractory (r/r) B-ALL (ABCs)

*FDA* Food and Drug Administration, *EMA* European Medicines Agency

## Conclusion

Immunotherapeutic agents are preferred over standard chemotherapy for patients with relapsed or refractory B-ALL. Nevertheless, there are no efficacy or safety head-to-head studies comparing these agents. The combination or sequence of these different immunotherapy options over the course of treatment remain a great challenge. Future studies may uncover biomarkers that could drive a more individualized approach to address effectiveness and prevent mechanisms of resistance.
